# Prediction of the CYP2D6 enzymatic activity based on investigating of the CYP2D6 genotypes around the vivax malaria patients in Yunnan Province, China

**DOI:** 10.1186/s12936-021-03988-5

**Published:** 2021-11-25

**Authors:** Ying Dong, Herong Huang, Yan Deng, Yanchun Xu, Mengni Chen, Yan Liu, Canglin Zhang

**Affiliations:** 1 Yunnan Institute of Parasitic Diseases Control, Yunnan Provincial Key Laboratory of Vector-Borne Diseases Control and Research, Yunnan Centre of Malaria Research, Pu’er, 665000 China; 2 Department of Basic Medical Sciences, Clinical College of Anhui Medical University, Hefei, 230031 China

**Keywords:** Yunnan Province, Vivax malaria patient, CYP2D6, Genotype, Enzyme activity, Prediction

## Abstract

**Background:**

In recent years, the incidence rate of vivax malaria recurrence still had 3.1% in Yunnan Province population after eradication therapy using primaquine (PQ). In order to understand the specific failure reasons for preventing vivax malaria relapses, a preliminary exploration on the CYP2D6 enzyme activity was carried out in the vivax malaria patients in Yunnan Province population by analysing mutational polymorphism in the coding region of CYP2D6 gene.

**Methods:**

Blood samples were collected from vivax malaria patients with suspected relapse (SR) and non-relapsed (NR) malaria in Yunnan Province. The DNA fragments containing 9 exons regions of human CYP2D6 gene were amplified by performing PCR and sequenced. The sequencing results were aligned by using DNAStar 11.0 to obtain the coding DNA sequence (CDS) of CYP2D6 gene. DnaSP 6.11.01 software was used to identify mutant polymorphisms and haplotypes of the CDS chain. The waterfall function of GenVisR package in R was utilized to visualize the mutational landscape. The alleles of CYP2D6 gene were identified according to the criteria prescribed by Human Cytochrome P450 (CYP) Allele Nomenclature Committee Database and the CYP2D6 enzyme activity was predicted based on diploid genotype.

**Results:**

A total of 320 maternal CDS chains, including 63 from SR group and 257 from NR group, were obtained. Twelve mutant loci, including c.31 (rs769259), c.100 (rs1065852), c.271 (rs28371703), c.281 (rs28371704), c.294 (rs28371705), c.297 (rs200269944), c.336 (rs1081003), c.408 (rs1058164), c.505 (rs5030865), c.801 (rs28371718), c.886 (rs16947), and c.1,457 (rs1135840) were observed on the 640 CDS chains (including 320 maternal and 320 paternal chains). The high-frequency mutation at rs1135840 (0.703) and low-frequency mutation, such as rs28371703, were detected only in the SR group. The frequency of mutant rs1058164 and rs1135840 were significantly increased in the SR group (${x}^{2}$= 4.468, 5.889, *P* < 0.05), as opposed to the NR group. Of the 23 haplotypes (from Hap_1 to Hap_23), the nomenclatures of 11 allelic forms could be found: Hap_3 was non-mutant, Hap_2 accounted for the highest frequency (36.9%, 236/640), and Hap_9 had the most complex sequence structure, containing 7 loci mutations. Allele *10 was the most frequent among these genotypes (0.423). Among the allele *10 standard named genotypes, *1/*10, *1/*1 and *2/*10 were significantly more frequent in the NR group (${x}^{2}$= 3.911, *P* < 0.05) and all showed uncompromised enzyme activity; the impaired genotype *10/*39 was more frequent in the SR group (${x}^{2}$= 10.050, *P* < 0.05), and genotype *4/*4was detected only in the SR group.

**Conclusion:**

In the patients receiving PQ dosage in Yunnan Province population, both rs1135840 single nucleotide polymorphism and *10 allele form was common in the CYP2D6 gene. Low-frequency mutation sites, such as rs28371703, were only presented in patients with vivax malaria relapse.

**Supplementary Information:**

The online version contains supplementary material available at 10.1186/s12936-021-03988-5.

## Background

PQ currently remains the only reliable drug to eliminate the hypnozoites of *Plasmodium vivax* [1]. However, the production of its active ingredient, 5-hydroxy-primaquine, relies on the catalysis of CYP2D isoenzymes in human hepatocytes [2–4]. CYP2D6 is the only monooxygenase in the P450 family that cannot be induced by the environment. CYP2D6 is characterized by low capacity, high catalytic efficiency, and complex heterogeneity; more than 30% of commonly used clinical drugs are catalysed by CYP2D6 [4–6]. Previous study has revealed the association between CYP2D6 gene polymorphism and the ineffective potency of PQ in the radical cure of vivax malaria [7, 8]. Evidence revealed the notably high frequency of rs1065852 and rs1135840 locus mutations with vivax malaria relapse patients in a Brazilian population [7, 9].

In relapsed vivax malaria cases in Papua New Guinea, CYP2D6 enzyme activity was mostly deficient star alleles *3, *4, *5, *6, *7, and *8, which indicates the phenotype of poor metabolizer (PM) [10, 11]. However, the impaired degree of CYP2D6 enzyme activity induced by non-functional star allele *3, homozygous diploid *3/*3 [12], as well as the dose–effect relationship between the copy number and the relapses occurring vivax malaria patients [7, 8] remains to be clarified.

Recently, the World Health Organization (WHO) classified the impaired CYP2D6 function as one of the four risk factors for PQ therapy, in addition to G6PD deficiency, pregnancy and/or lactation and infancy [1, 13, 14]. The WHO further advocated the personalized dosage of PQ therapy for the eradicative treatment of vivax malaria based on acquired understanding of patients’ CYP2D6 genetic polymorphism and heterogeneity of enzyme activity [14, 15].

Molecular epidemiological investigation on G6PD deficiency in vivax malaria patients has been initiated in Yunnan Province [16, 17] to carry out polymorphism analysis of the full coding region of G6PD gene, revealing that G > T (rs72554665) missense mutation at c.1376 locus could be used as the molecular marker for indicating G6PD deficiency. It also found that vivax malaria patients with genotype *4/*4 of CYP2D6 poor enzyme activity were prone to the failure of PQ radical treatment for vivax malaria [7], suggesting that the influence of biological factors may cause obstacles to effectively block the transmission of vivax malaria and effectively eliminate sources of malaria infection in Yunnan Province. The current study further analysed CYP2D6 genotype and its predicted enzyme activity in vivax malaria patients from Yunnan Province population who underwent PQ treatment in order to systematically understand factors that may adversely affect deployment of PQ for radical cure of vivax malaria in Yunnan Province.

## Methods

### Study subjects and blood sampling

All vivax malaria cases included in the current study had been diagnosed and reported by county-level laboratories in Yunnan Province since 2014. The initial diagnosis from county-level laboratories were re-tested by Yunnan Provincial Malaria Diagnostic Reference Laboratory (YPMDRL) using both microscopic examination and genetic testing (Additional file 1) of *Plasmodium* in peripheral blood [18]. YPMDRL was formally certified as a member of the China Malaria Diagnosis Reference Laboratory Network in 2012 [19], which is composed of provincial microscopists and molecular biology experts. Based on collaborative relationship between YPMDRL and 129 county-level malaria diagnosis laboratories, every malaria case reported from Yunnan Province was accurately identified [20]. During the period, China had implemented a malaria epidemic reporting and review system to ensure the integrity of all malaria cases diagnosed and reported since 2005. The peripheral blood samples from these cases had been collected in YPMDRL since 2014. The 0.6 ml of venous blood collected from every subject was stored in a dried tube at − 80 °C before DNA extraction. Dried blood spot (DBS) sampling was used to help with genetic analysis.

Anti-malarial treatment for malaria cases was carried out in county-level hospitals after completing initial diagnosis. All vivax malaria patients received oral chloroquine therapy (1550 mg in total) within 3 days, followed by the subsequent 8-day course of primaquine therapy (22.5 mg/day) (commonly known as 8-day chloroquine/primaquine therapy). The vivax malaria recurrence cases were found by the county-level laboratories, too. Everyone received 8-day chloroquine/primaquine therapy was able be identified as recurrence case when his peripheral blood showed *Plasmodium* infection again after 28 days under no longer had the experience of exposure to malaria [18].

Then, it is necessary that the paired infection strains between previous and recurrence episodes were molecularly identified by YPMDRL after that. The paired strains, between the previous and recurrent infections, were molecularly identified by YPMDRL, and verified as the single clone with homologous or the sibling clones with weak heterologous DNA sequences including both *pvcsp* gene and *pvmsp-1* gene [18, 21] (Published in another manuscript: Molecular identification of vivax malaria recurrent episodes in Yunnan Province basing on analysis of the homology between *pvcsp* genes in different *Plasmodium vivax* stains), the case with recurrent experience can be confirmed as vivax malaria suspected-relapse.

In order to systematically reveal the heterogeneity of CYP2D6 enzymatic activity in vivax malaria cases in Yunnan Province, the current study adopted a grouping and cluster sampling survey. The vivax malaria patients were divided into suspected-relapse group (SR group) and non-relapse group (NR group). The NR group, which included the cluster patients from 2018 to 2020, had not experienced recurrent symptoms after being clinically cured by 8-day chloroquine/primaquine therapy. In the SR group, all patients from 2014 to 2020 were included.

### Extraction of human genomic DNA and PCR amplification of CYP2D6 gene fragments

The DBS method was used on filter paper dried blood stain (diameter = 5 mm). QIAgen Mini Kit (DNA Mini Kit, QIAamp, Germany) was used to extract human genomic DNA from the blood stain, according to manufacturer’s instructions. DNA samples were stored at -20 °C for subsequent assay.

Reference sequence (ID: NC_000022.11) was used as the template to design the PCR primers and to determine the reaction conditions for CYP2D6 gene, according to methods described in previous studies [18, 22, 23]. The coding regions covering exons 1–4 and exons 5–9 were amplified by segmentation. It is predicted that the amplification regions contain from 42131088 to 42128678 and from 42126035 to 42128422 and amplification products with length of 2411 bp and 2388 bp, respectively. The amplification products were sequenced by Shanghai Meiji Biomedical Technology Co. Ltd, using the Sanger method.

### Analysis of CYP2D6 gene coding region polymorphism and prediction of enzyme activity

The sequencing results were collated by using DNAStar 11.0 and BioEdit 7.2.5, and the first coding DNA sequence (CDS) chain of CYP2D6 gene of each sample, known as maternal CDS chain, was obtained by the splicing method [18]. The maternal CDS chain was used to derive the paternal CDS chain by replacing the bases of the unread double allelic heterozygous sites [24], which were identified by searching the DNA sequencing peak chromatograms. DnaSP 6.11.01 software was used to identify site-specific mutations and haplotypes in the CDS chain, to calculate the nucleic acid diversity index (π) and expected heterozygosity (He), which were aligned with referent sequence and determined the SNP ID of each mutation on the National Center for Biotechnology Information (NCBI) platform (https://www.ncbi.nlm.nih.gov/snp/). GenVisR package in R (version: 4.0.2) was used to visualize the mutational waterfall plots of the haplotypes of the loci in the allelic form.

The haplotypes (allelic forms) of the CDS chain of CYP2D6 gene were determined in accordance with the criteria of the Human Cytochrome P450 Allele Nomenclature Committee Database (NM_000106.5) [25] and published literature [18, 26]. The sub allelic forms can be directly defined by haplotype. Haplotypes devoid of criteria for allelic forms were customized as * + a single lowercase letter [18].

The genotype of each sample was presented in the diploid form consisting of maternal and paternal CDS chains, such as *10/*39, *1/*10 and so forth. The diploid allelic form of the genotype was no longer refined to the sub-allelic level, as the sub-allelic forms should be merged in the first place. For example, *10.001 and *10.002 could be combined into the allelic form *10 to count the frequency.

For the genotypes with standard nomenclature, the CYP2D6 enzyme activity was predicted by calculating the genotype score based on diploid genotypes, following the previously described method [11, 27, 28]. The genotype score is the sum of two allelic form scores. For instance, a score of 0.75 for genotype *10/*39 is the sum of 0.25 score for allelic form *10, and 0.5 score for allelic form *39. In the event of genotype score being 0.0, the enzyme activity is absent and hence poor metabolizer (PM). The genotype score ranging from 0.25 to 1.0, 1.25 to 2.25, and > 2.25 indicates intermediate metabolizer (IM), normal metabolizer (NM), and ultrarapid metabolize (UM), respectively [11, 22, 23, 29–31]. Meanwhile, the online tool PROVEAN (http://provean.jcvi.org/index.php) was used to predict the function of CYP2D6 enzyme protein of different genotypes as deleterious (DT) or neutral (NT).

### Statistics

The frequency of mutation site was presented by the ratio of the number of base substitutions to the total number of CDS chains. Each study subject had two CDS chains, including maternal chain and paternal chain. Chi-square test $\left( {x^{2} } \right)$ was used to analyse the differences in locus mutations and genotypes between the two groups. *P* < 0.05 was regarded statistically significant.

## Results

### Sample information and PCR amplification of CYP2D6 gene fragment

A total of 345 blood samples were collected from vivax malaria patients for subsequent PCR amplification of human genomic DNA, which covers exon1-4 and exon5-9 regions of CYP2D6 gene. PCR amplification products showed the target fragment more than 2000 bp bands at gel electrophoresis. After collating the sequencing results of PCR amplification products, all 320 maternal CDS chains of CYP2D6 gene (1491 bp in length) were obtained in 345 subject blood samples (92.8%, 320/345), including 63 SR cases and 257 NR cases.

The blood samples of obtained 320 maternal CDS chains were collected from diagnosed vivax malaria cases in the following prefectures of Yunnan Province: Yingjiang (182), Mangshi (15), Longchuan (8), Ruili (16), Tengchong (42), Baoshan (20), Lianghe (3), Mengla (1), Menglian (1), Diqing (1), Nujiang (1), Jinping (4), Yuanyang (2), Lincang (20), Dali (2) and Kunming (2).

The demographic and clinical characteristics of 320 cases are listed in Table 1 and Additional file 2. The male to female ratio was 2.6:1. Most of the vivax malaria patients were imported into Yunnan Province after infection in Myanmar (98.4%, 315/320) and presented one suspected relapsed episode (95.2%, 60/63) (Table 1).

**Table 1:** Information of 320 vivax malaria cases for amplification of CYP2D6 gene CDSs

Variable	Total (n, F%)	SR group (n, F%)	NR group (n, F%)
Total	320 (100.0)	63 (19.7)	257 (80.3)
1. Gender			
Male	233 (72.8)	46 (73.0)	187 (72.8)
Female	87 (27.2)	17 (27.0)	70 (27.2)
2. Age (in years)			
0–4	6 (1.9)	2 (3.2)	4 (1.6)
5–20	36 (11.3)	3 (4.8)	33 (12.8)
21–60	260 (81.2)	54 (85.7)	206 (80.2)
Above 60	18 (5.6)	4 (6.3)	14 (5.4)
3. Malaria relapse			
1 episode	60 (18.8)	60 (95.2)	–
2 episodes	2 (0.7)	2 (3.2)	
3 episodes	1 (0.3)	1 (1.6)	–
4. Infection source ^a^			
Myanmar	315 (98.4)	60 (95.2)	255 (99.2)
Africa	3 (0.9)	1 (1.6)	2 (0.8)
Yunnan indigenous	2 (0.7)	2 (3.2)	–
5. Days of recurrence (d)			
Shortest	–	29	–
Median		171	
Longest	–	1605	–

*n* number of cases, *F* Frequency

^a^Identified by epidemiological investigation

### CDS chain polymorphism and allelic forms of CYP2D6 gene

The polymorphism of 640 CDS chains, 320 maternal and 320 paternal, of CYP2D6 genes revealed base substitutions (G > A, C > T, C > A.T, A > G, C > G, C > T, C > T, G > C, G > A, C > A, C > T and G > C) at 12 loci, includingc.31, c.100, c.271, c.281, c.294, c.297, c.336, c.408, c.505, c.801, c.886, and c.1,457. Most of these mutation sites have been registered as single nucleotide polymorphism (SNP) loci (Table 2). Of these loci, c.31 (rs769258), c.100 (rs1065852), c.271 (rs28371703), c.281 (rs28371704), c.505 (rs5030865), c.886 (rs16947), and c.1,457 (rs1135840) were non-synonymous mutations. On the amino acid peptide chain, the 11th, 34th, 91th, 94th, 169th, 296th, and 486th amino acids exhibited V/M, P/S, L/M, H/R, G/S, R/C, and S/T variants (Table 2).

**Table 2:** The polymorphism and diploid of CDS mutation loci in CYP2D6 gene of vivax malaria cases in Yunnan Province

Loci	SNP ID^a^	Codon change	Amino acid change	Frequency ^b^	Diploid				Different type of cases		
					No. loci	NMHO ^c^ (CR) ^d^	MHE ^e^ (CR) ^d^	MHO ^f^ (CR) ^d^	No. SR(TPR)^g^	No. NR (TPR)^g^	*P*
c.31	rs769258	GTG > ***A***TG	V11M	0.002	1	319 (99.7)	1 (0.3)	0 (0.0)	1 (1.6)	0 (0.0)	0.197
c.100	rs1065852	CCA > ***T***CA	P34S	0.527	337	87 (27.2)	129 (40.3)	104 (32.5)	43 (68.3)	190 (73.9)	0.364
c.271	rs28371703	CTG > ***A***TG	L91M	0.006	4	318 (99.4)	0 (0.0)	2 (0.6)	2 (3.2)	0 (0.0)	0.038 h
		CTG > ***T***TG	L91L	0.003	2	319 (99.7)	0 (0.0)	1 (0.3)	0 (0.0)	1 (0.4)	1
c.281	rs28371704	CAC > C***G***C	H94R	0.006	4	318 (99.4)	0 (0.0)	2 (0.6)	2 (3.2)	0 (0.0)	0.038 h
c.294	rs28371705	ACC > AC***G***	T98T	0.006	4	318 (99.4)	0 (0.0)	2 (0.6)	2 (3.2)	0 (0.0)	0.038 h
c.297	rs200269944	GCC > GC***T***	A99A	0.002	1	319 (99.7)	1 (0.3)	0 (0.0)	1 (3.2)	0 (0.0)	0.197
c.336	rs1081003	TTC > TT***T***	F112F	0.552	353	93 (29.1)	101 (31.5)	126 (39.4)	39 (61.9)	188 (73.2)	0.078
c.408	rs1058164	GTG > GT***C***	V136V	0.698	447	54 (16.9)	85 (26.6)	181 (56.5)	58 (92.1)	208 (80.9)	0.035 h
c.505	rs5030865	GGT > ***A***GT	G169S	0.003	2	318 (99.4)	2 (0.6)	0 (0.0)	2 (3.2)	0 (0.0)	0.038 h
c.801	rs28371718	CCC > CCA	P267P	0.002	1	319 (99.7)	1 (0.3)	0 (0.0)	0 (0.0)	1 (0.4)	1
c.886	rs16947	CGC > ***T***GC	R296C	0.178	114	220 (68.8)	86 (26.9)	14 (4.4)	24 (38.1)	76 (29.6)	0.191
c.1457	rs1135840	AGC > A***C***C	S486T	0.703	450	46 (14.4)	98 (30.6)	176 (55.0)	60 (95.2)	214 (83.3)	0.015 h

^a^The SNP ID was determined by searching on NCBI platform

^b^The denominator was 640, which is the total number of 320 maternal CDS chains and 320 paternal CDS chains

^c^Constituent ratio, the denominator was 320

^d^*NMHO* not mutation homozygote

^e^*MHE* mutation heterozygote

^f^*MHO* mutation homozygote

^g^*TPR* test positive rate

^h^Chi-square test (significant level at P < 0.05)

The highest frequency of locus mutation was observed at c.1,457 (70.3%, rs1135840), and the minor allele frequency (MAF) was 0.698 for c.408 (rs1058164) (Table 2). Loci mutation at c.271 (rs28371703), c.281 (rs28371704), c.294 (rs28371705), and c.505 (rs5030865) were detected in only the SR group. Meanwhile, mutation at c.408 (rs1058164) and c.1457 (rs1135840) showed significantly higher frequency in the SR group (x^2 = 4.468, 5.889, P < 0.05), as opposed to the NR group (Table 2). Mutant homozygotes were mainly composed of four loci, including c.408 (rs1058164), c.1,457 (rs1135840), c.336 (rs1081003), and c.100 (rs1065852) (Table 2).

**Fig. 1:**
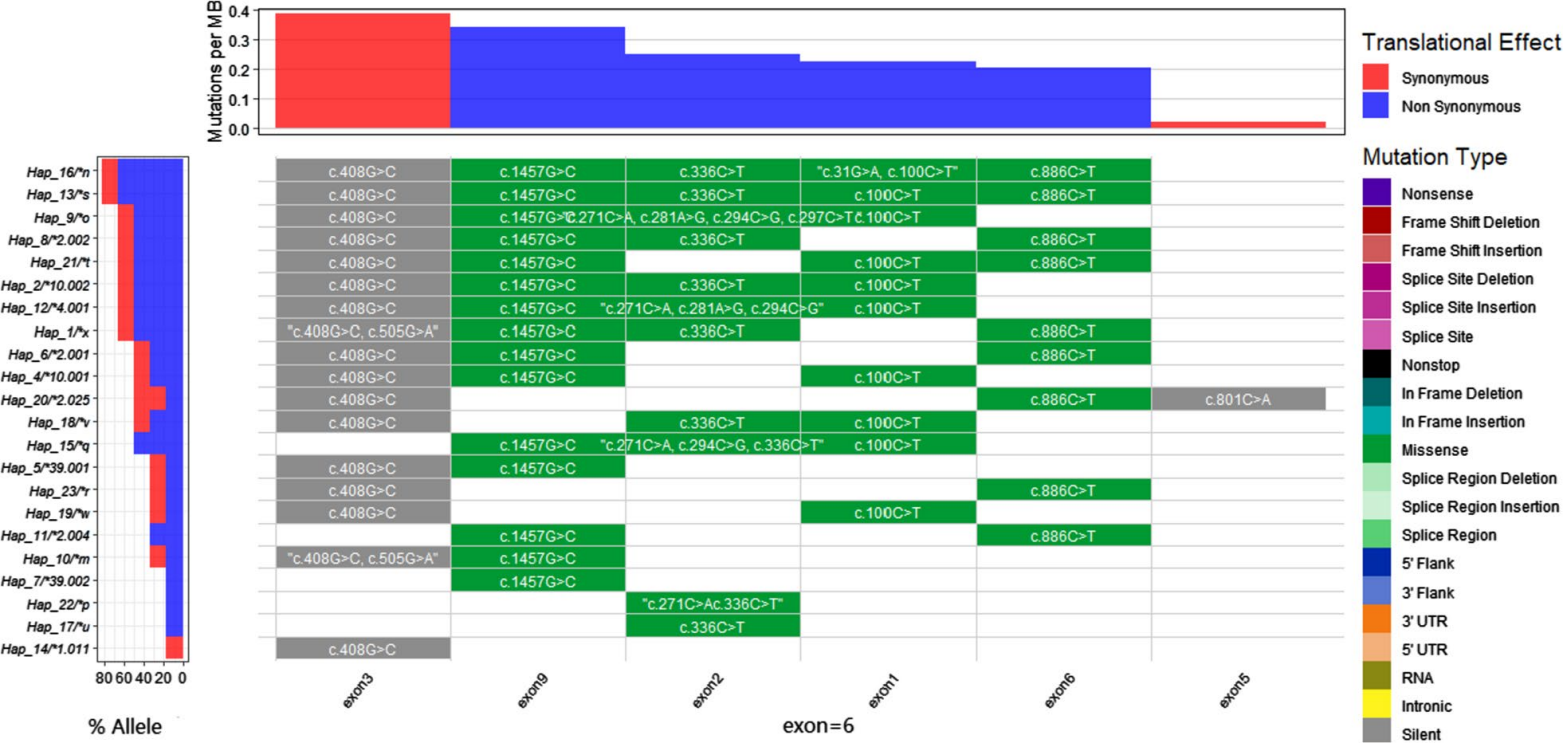
Waterfall plotting of mutation loci in different haplotypes (allelic form) in the CDS chains of CYP2D6 gene

These 640 CDS chains could be subdivided into 23 haplotypes (from Hap_1 to Hap_23, π = 0.002, He = 0.772) (Fig. 1). Among them, the non-mutant Hap_3 accounted for 26.6% (170/640). In contrast, Hap_1, Hap_2, and Hap_4 to Hap_23 were mutant, which accounted for 0.2% (1/640), 36.9% (236/640) and 9.1% (58/640), 4.5% (29/640), 10.2% (65/640), 0.6% (4/640), 0.8% (5/640), 0.2% (1/640), 0.3% (2/640), 1.1% (7/640), 0.5% (3/ 640), 3.6% (23/640), 0.9% (6/640), 0.2% (1/640), 0.2% (1/640), 0.2% (1/640), 1.3% (8/640), 1.3% (8/640), 0.5% (3/640), 0.3% (2/640), 0.6% (4/640), 0.2% (1/640), and 0.3% (2/640), respectively.

Of the 23 haplotypes, Hap_2, Hap_3, Hap_4, Hap_5, Hap_6, Hap_7, Hap_8, Hap_11, Hap_12, Hap_14, and Hap_20, were known haplotypes and could be identified as *10.002, *1.001, *10.001, * 39.001, *2.001, *39.002, *2.002, *2.004, *4.001, *1.011, and *2.025 in the respective allelic forms. The remaining 12 haplotypes were presented in the allelic form of *m ~ *x (Fig. 1).

When compared with the non-mutated sequence (Hap_3), Hap_9 showed the most complicated sequence composition, exhibiting mutations sites at c.100, c.271, c.281, c.294, c.297, c.408, c.1,457 and so forth. Hap_14 showed the simplest sequence composition, with mutation detected at c.408 (Fig. 1). On the other hand, the exon 3 was the region with the highest mutation frequency,73.9% (17/23) in 23 haplotypes, but the heat of mutation in exon 9, exon2, exon 1, exon 6, and exon 5 decreased gradually (Fig. 1), showing the loci mutation frequency of 65.2% (15/23), 47.8% (11/23), 43.5% (10/23), 39.1% (9/23), and 4.5% (1/23), respectively (Fig. 1). In contrast, no locus mutations could be determined in exons 4, 7 and 8.

### CYP2D6 genotypes and enzyme activity

A total of 34 CYP2D6 genotypes were obtained from blood samples of 320 vivax malaria patients. Among them, genotypes*1/*1, *2/*2, *1/*2, *2/*10, *1/*10, *10/*10, *10/*39, *39/*39, *2/*39, and *4/*4 belong to the named genotypes (Table 3), and are suitable for assignments of allelic form and prediction of CYP2D6enzyme activity. These variants jointly accounted for 84.1%in the study population (269/320). Of all genotypes, *10/*10 was the most frequent (26.9%, 86/320), with the remaining genotype *1/*10* being 18.1%(58/320)and *1/*1being 13.1% (43/320) (Table 3). The genotypes of 15.9% (51/320) of patients could not be determined due to failure of the assay to identify one or both allelic. Among 10 documented genotypes, the most common was *10/*10, a predicted IM phenotype, and by the PROVEAN method, NT. In spite of the predicted reduced enzyme activity, the rate of relapse between SR and NR groups was not statistically significant (${x}^{2}$ = 0.115, *P* > 0.05) (Table 3). The other genotypes with frequent mutation included *1/*10, *1/*1 and *2/*10 (10.0%, 32/320), which were predicted as NM and NT by both genetic method and PROVEAN methods. The frequency of these three allelic variants was all significantly higher in the NR group (${x}^{2}$ = 3.911, 7.103 and 4.060, *P* < 0.05) than in the SR group (Table 3). In contrast, genotypes *10/*39 (2.8%, 9/320), manifesting impaired enzyme activity (IM and DT), were predicted by both two methods, with frequency being significantly higher in the SR group (${x}^{2}$ = 10.050, *P* < 0.05), compared with the NR group (Table 3). Genotype *4/*4 indicating deficient enzyme activity (PM and DT) was found only in the SR group (Table 3).

**Table 3:** Prediction of CYP2D6 enzyme activity based on genotyping of CDS chain of vivax malaria patients in Yunnan Province

Genotypes	No (CR)^a^	Genotype mothed		PROVEAN mothed			Different type of cases		
		Value	Classify	Score	Cutoff	Function	No. SR (TPR)^b^	No. NR (TPR)^b^	*P*
*1/*1	43 (13.4)	2.0	NM	2.000	− 2.5	NT	2 (3.2)	41 (16.0)	0.008^c^
*2/*2	10 (3.1)	2.0	NM	− 0.887	− 2.5	NT	4 (6.3)	6 (2.3)	0.216
*1/*2	22 (6.9)	2.0	NM	− 0.773	− 2.5	NT	7 (11.1)	15 (5.8)	0.228
*2/*10	32 (10.0)	1.25	NM	− 1.568	− 2.5	NT	2 (3.2)	30 (11.7)	0.044^c^
*1/*10	58 (18.1)	1.25	NM	− 2.341	− 2.5	NT	6 (9.5)	52 (20.2)	0.048 ^c^
*10/*10	86 (26.9)	0.50	IM	− 1.671	− 2.5	NT	18 (28.6)	68 (26.5)	0.735
*10/*39	9 (2.8)	0.75	IM	− 2.666	− 2.5	DT	6 (9.5)	3 (1.2)	0.002^c^
*39/*39	3 (0.9)	1.0	IM	1.350	− 2.5	NT	2 (3.2)	1 (0.4)	0.100
*2/*39	5 (1.6)	1.50	NM	− 1.098	− 2.5	NT	3 (4.8)	2 (0.8)	0.054
*4/*4	1 (0.3)	0	PM	− 9.431	− 2.5	DT	1 (1.6)	0 (0.0)	0.197
Others ^d^	51 (15.9)	–	–	–	–	–	12 (19.0)	39 (15.2)	0.452
Total	320						63	257	

^a^CR is acronym for constituent ratio

^b^TPR is acronym for Test positive rate

^c^Chi-square test (significant level at *P* < 0.05)

^d^The diploids with at least one undocumented allele (*m, *n, *o, *p, *q,*r, *s,*t, *u, *v, *w and *x)

## Discussion

Located in the non-telomeric region of the long arm of human chromosome 22, CYP2D6 gene has 4,383 bp in length and is composed of 8–9 exons and 7–8 introns. The CDS chain of CYP2D6 has 1,338–1,491 bp to encode 446–497 amino acids. To be more specific, CYP2D6 gene with 9 exons has an inserted sequence encoding 51 amino acids chains of NVFLARYGPAWREQRRFSVSTLRNLGLGKKSLEQWVTEEA.

ACLCAAFANHSGC in the downstream of exon2, compared with that has 8 exons by aligning sequence [32]. The CYP2D6 genes analysed in this study all have 9 exons, and as high as 92.8% of the maternal CDS chains were obtained. Such a high efficiency of PCR amplification of CYP2D6 gene could be attributable to the absence of Poly-T/A structure in the CYP2D6 gene and the lower degree of DNA polymerase slippage on the template to facilitate the extension of DNA chain.

To date, as many as 300 allelic and sub-allelic variants of CYP2D6 gene have been identified [25]. Although the 12 locus mutations detected in this study at the CYP2D6 locus, such as c.31 (rs769259), c.100 (rs1065852), c.886 (rs16947), and c.1,457 (rs1135840), were all validated SNPs, up to 52.2% (12/23) of the star allelic variants by combination [32] have not been documented or assigned [24–27]. This could hinder accurate classification of CYP2D6 enzyme activities based on the genotype score of biallelic mutation for prediction. The insensitivity in the previous confirmation criteria of allele [25, 26] to the combination of mutant loci obtained by whole gene sequencing is the most probable reason, in addition to the lack of accuracy due to the inclusion of intron SNPs in CYP2D6 gene allele identification.

Intriguingly, low-frequency mutant loci, such as c.271 (rs28371703), c.281 (rs28371704), c.294 (rs28371705), and c.505 (rs5030865), were present only in samples of SR group (Table 2). This study observed that genotype *4.001 and diploid *4/*4indicate null CYP2D6 enzyme function (PM), a conclusion consistent with the findings of Bennett et al. [2], who validated that allele *4 signifies severely impaired CYP2D6 enzyme activity. Although the frequency of homozygous diploid *4/*4 in the population of vivax malaria patients in Yunnan Province was 0.003 (1/320), which is much lower than 0.12 (27/220) in Brazilians [33], and 0.008 (2/250) in Whites [34]. However, Silvino’s study showed that the most common CYP2D6 diploid from vivax malaria recurrence patients were predicted as reduced metabolism *2/*4[34], indicating that allele *4 would have a negative effect on CYP2D6 enzyme activity. Thus, the rational suggestion put forward has been that those mutations at rs28371703, rs28371704, rs28371705 as well as other loci may be applied as the molecular markers of impaired CYP2D6 enzyme activity in clinical practice. Mutant variant rs16947, which was validated as damaged CYP2D6 enzyme activity in a previous study [35], showed a frequency of merely 17.8% in the present study. However, the allelic form *2 and its homozygous diploid *2/*2 did not display preferential distribution in the SR group or the NR group, let alone presaging the phenotype of abnormal enzyme activity (Table 3). This could be attributed to the rigorous scoring and classification of CYP2D6 enzyme activity, which could not reflect the subtle disparities in the genetic structure.

In contrast, the PROVEAN method, by taking every amino acid substitution into the CYP2D6 peptide chain rather than calling the known allelic combination like scoring method preserves the full amount of polymorphic information of the gene structure in its prediction results. Furthermore, in this study, the reduced activity allelic form *10 was detected in 57.8% (185/320) of the samples [36, 37], and exhibited the highest frequency (0.423) in the samples. This is consistent with the previous conclusion that allele *10 is more widely distributed in Southeast Asian populations [38], yet the notable preferential distribution of genotype *10/*10 cannot be found, either in the SR group or the NR groups. In contrast, genotype *10/*39 indicates both IM and DT, and was commonly observed in the SR population. It is puzzling that predicted enzyme activity phenotype level of homozygous diploid *39/*39 by PROVEAN method is not lower or the same as genotype *10/*39. The speculated reason may be related to different allelic by combined inappropriately from some loci. For example, both allele *2.001and allele *10.001 were composed of three loci mutations at c.408, c.1,457, c.886 and at c.408, c.1,457, c.100 (Fig. 1), respectively, while the allele score of the former was higher than the latter. In addition to other Southeast Asian countries, the allele *39 and homozygous diploid *39/*39 were found in Yunnan Province, suggesting that allele *39 was not an accidental existence in the local population although the 0.03 frequency of allele *39 was lower than 0.2 in central/South Asia and East Asia [39]. Such findings remind that during the process of effectively eliminating the infectious source of imported vivax malaria, Yunnan Province should pay attention to the effect of allele *39 existence, which may be one of the biological factors affecting eradication of malaria with PQ. Moreover, although the recent study shows that genotype *10/*39 has been a risk factor affecting PQ efficacy, whether sequencing CYP2D6 gene for PROVEAN assessment or not, it still needs to be selected according to the roles of different laboratories. After all, when there are residual blood samples of cases exposed to PQ, it is no technical obstacle to supply the sequencing analysis of CYP2D6 gene, and it will not bring any negative impact on these cases’ health. However, the heterogeneity of CYP2D6 enzymatic activity predicted only by molecular markers cannot be used as the basis for adjusting drug policy on PQ in Yunnan Province. The authors promoted the testing of substrate metabolic activity from different CYP2D6 genotypes according to previous standard [12, 30] to observe the phenotype of CYP2D6 enzyme activity.

The radical cure of vivax malaria is a critically important intervention to prevent the re-transmission and repeated attack of malaria [38]. As we know, the study of the CYP2D6 gene allelic polymorphism and its distribution in the PQ- exposed population in Yunnan Province was firstly reported, and the experimental approaches are developed and introduced to press for the accurate screening of population suitable for PQ therapy in Yunnan Province.

This study has certain shortcomings. First, the determination of the recurrence of vivax malaria was somewhat flawed, as the *Plasmodium* reservoir in bone marrow into peripheral blood [40, 41] or the resistance of *Plasmodium* to PQ [42], despite efforts to validate the genetic consistency of the infected strains of patients with recurrent vivax malaria. Second, the effect of gene copy number on the prediction of CYP2D6 enzyme activity was not ruled out. It was shown that once the normal allele of CYP2D6 gene activity has more than two copies, CYP2D6 enzyme activity will be changed from the phenotype of NM into UM [43, 44]. Third, SNPs or single nucleotide variant (SNV) in the intron region of CYP2D6 gene were not identified nor applied to define the allelic form, hence more undocumented allelic forms of CYP2D6 gene require further validation. In the next stage, the research team will refine and optimize the experimental protocol by making constant adjustment.

## Conclusions

In this study, only 12 mutation loci, including c.31, c.100, c.271, c.281, c.294, c.297, c.336, c.408, c.505, c.801, c.886, and c.1,457 were detected in Yunnan Province vivax malaria patients, which is consistent with the findings of a previous small sample. In those patients receiving PQ dosage in Yunnan Province, both rs1135840 SNP and allele form *10 was common in the CYP2D6 gene coding region. Low-frequency mutation sites, such as rs28371703, were presented only in patients with vivax malaria relapse. Genotype *10/*39 could be suitable for indicating impaired CYP2D6 enzyme activity in this population to be treated with PQ.

## Supplementary Information

**Additional file 1**. The details of nested PCR testing for differentiating between various Plasmodium species.

**Additional file 2**. Records of basic personal information of subjects

## Data Availability

Not applicable.

## Abbreviations

WHO: World Health Organization
CYP2D6: Cytochrome P450, family 2, subfamily D, polypeptide 6
G6PD: Glucose-6-Phosphate Dehydrogenase
CDS: Coding DNA sequence
PCR: Polymerase chain reaction
YPMDRL: Yunnan Province Malaria Diagnosis Reference Laboratory
DBS: Dried blood spots
*pvcsp*: *Plasmodium vivax* Circumsporozoite surface protein
*pvmsp-1*: *Plasmodium vivax* Merozoite surface protein 1
Hap: Haplotype
π: Diversity index
He: Expected heterozygosity
CI: Confidence interval
SNP: Single nucleotide polymorphism
SNV: Single nucleotide variant
PM: Poor metabolizer
IM: Intermediate metabolizer
NM: Normal metabolizer
UM: Ultrarapid metabolizer
DT: Deleterious
NT: Neutral
MAF: Minor allele frequence
PQ: Primaquine
NCBI: National Center for Biotechnology Information
