# Supplementary material for: Prediction of the CYP2D6 enzymatic activity based on investigating of the CYP2D6 genotypes around the vivax malaria patients in Yunnan Province, China
Source: Malar J. 2021 Nov 25;20:448. doi: 10.1186/s12936-021-03988-5 (PMC8620920; doi:10.1186/s12936-021-03988-5)
Supplement: Supplementary file 1 — Additional file 1. The details of nested PCR testing for differentiating between various Plasmodium species. [file 12936_2021_3988_MOESM1_ESM.docx]

| **Additional file 1 The details of nested PCR testing for differentiating between various *Plasmodium* species** | | | | | | |
| --- | --- | --- | --- | --- | --- | --- |
| Nested PCR | Specificity of primers | Primer name^△^ | Primer sequence^△^ | Expected PCR product (bp) | Reaction conditions | Reaction systems |
| First round | Genus | rPLU5 | 5’-CCTGTTGTTGCCTTAAACTTC-3； | 1200 | 94 ° C for 3min;94° C for 30s, 58° C for 30s, 72° C for 60s, 34 cycles; 72° C for 5min. | 25 ul reaction volume including 2.6 μl template, 14.0 μl 2 × PCR Mix hybrid system (Containing Taq enzyme), 0.7 μl upstream primer (20umol / L) and 0.7 μl downstream primers (20umol / L) |
|  |  | rPLU6 | 5’-TTAAAATTGTTGCAGTTAAAACG-3’ |  |  |  |
| Second round | *P. falciparum* | rFAL1 | 5’-TTAAACTGGTTTGGGAAAACCAAATATATT-3’ | 205 | 94 ° C for 3min;94° C for 30s, 60° C for 30s, 72° C for 60s, 34 cycles; 72° C for 5min |  |
|  |  | rFAL2 | 5’-ACACAATGAACTCAATCATGACTACCCGTC-3’ |  |  |  |
|  | *P. vivax* | rVIV1 | 5’-CGCTTCTAGCTTAATCCACATAACTGATAC-3’ | 120 |  |  |
|  |  | rVIV2 | 5’-ACTTCCAAGCCGAAGCAAAGAAAGTCCTTA-3’ |  |  |  |
|  | *P. malariae* | rMAL1 | 5’-ATAACATAGTTGTACGTTAAGAATAACCGC-3’ | 141 |  |  |
|  |  | rMAL2 | 5’-AAAATTCCCATGCATAAAAAATTATACAAA-3’ |  |  |  |
|  | *P. ovale* | rOVA1 | 5’-ATCTCTTTTGCTATTTTTTAGTATTGGAGA-3’ | 800 |  |  |
|  |  | rOVA2 | 5’-GGAAAGGACACATTAATTGTATCCTAGTG-3’ |  |  |  |
| ^△^: The primers’ name and sequence were cited from References Snounou G, Viriyakosol S, Zhu XP, Jarra W, Pinheiro L, do Rosario VE, et al. High sensitivity of detection of human malaria parasites by the use of nested polymerase chain reaction. Mol Biochem Parasitol. 1993;61(2):315-20. | | | | | | |
